# Development of a new heat tolerance assay system for rice spikelet sterility

**DOI:** 10.1186/s13007-017-0185-3

**Published:** 2017-05-10

**Authors:** Makoto Hakata, Hiroshi Wada, Chisato Masumoto-Kubo, Ryo Tanaka, Hiroyuki Sato, Satoshi Morita

**Affiliations:** Kyushu Okinawa Agricultural Research Center, National Agriculture and Food Research Organization, 496 Izumi, Chikugo, Fukuoka 833-0041 Japan

**Keywords:** Spikelet fertility, Rice (*Oryza sativa* L.), Phytotron, High temperature, Heat tolerance, Flowering stage, Varietal difference

## Abstract

**Background:**

Reduction in rice yield caused by high temperature-induced spikelet sterility has been a serious concern in rice production. To date, several screening methods have been used, although their reproducibility is sometimes poor due to artifacts mainly caused by varietal differences in heading dates and panicle heights (i.e., the distance from the lamps).

**Methods:**

We have developed a novel assay system for heat-induced spikelet sterility by using artificial rice paddies in phytotrons to conduct a highly reproducible assay throughout a year. Plants restricted to the main culm were treated under a series of heat conditions, and height uniformity of each plant was ensured by using height-adjustable pots.

**Results:**

Results suggested that a 3-day heat treatment of 35 °C-day/29 °C-night cycles was the most suitable condition. Under the treatment, two distinct groups were identified among nine heat tolerant cultivars, with no varietal difference in panicle temperature, indicating that the system is capable of eliminating the varietal difference in panicle temperature.

**Conclusions:**

It is concluded that the assay system would be a powerful tool for selecting heat tolerant varieties, as well as the analysis of genetic factors from various cultivars, eliminating potential artifacts.

**Electronic supplementary material:**

The online version of this article (doi:10.1186/s13007-017-0185-3) contains supplementary material, which is available to authorized users.

## Background

At present, deterioration of rice quality caused by heat stress during ripening has been a serious concern in rice production owing to climate change [1–3]. However, as air temperatures increase, spikelet sterility caused by high temperatures is also expected to become a serious problem in rice production globally, including Japan, as simulated by Horie et al. [4]. In fact, severe heat-induced rice spikelet sterility caused by high temperatures during flowering was observed in the summer of 2003 in China [5]. As global warming advances, the instability of rice production will be enhanced owing to the increased frequency of heat-induced spikelet sterility [6].

Spikelet sterility caused by high temperatures is known to occur following heat exposure of panicles during booting and flowering [7]. It begins to occur when the daily maximum temperature reaches approximately 34–36 °C [8–10], and high humidity promotes spikelet sterility [10, 11]. Low humidity [8, 11], wind [11], and high CO_2_ concentration [11] also influence sterility. The mechanism(s) of rice spikelet sterility involves the lack of full development of pollen [12] and faulty dehiscence of anthers [7, 13] caused by high temperatures, resulting in a reduction in the number of healthy pollen grains at the stigma [7]. Consequently, heat-induced spikelet sterility causes a decline in fertility. However, the exact mechanism(s) of and the genes involved in heat-induced spikelet sterility remain unclear.

The impact on rice yield in Japan following further global warming using a crop simulation model suggested that the increasing high temperature tolerance by approximately 1.5 °C during the flowering period would greatly reduce the incidence of spikelet sterility [4, 14]. Therefore, the promotion of rice breeding of heat tolerant varieties is urgent, as well as understanding the mechanism(s) of high temperature-induced spikelet sterility, and the development of future adaptation technologies. To date, varietal differences in heat tolerance of spikelet sterility have been reported [9, 11, 13, 15–19]. The reported tolerant cultivars include three *Indica* cultivars, IR36, IR24, and IR64, two *Japonica* cultivars, Akitakomachi and Koshihikari, and an *aus* cultivar, N22. These cultivars were selected using three methods conducted under variable conditions, including temperature gradient chambers (TGC) under natural light, phytotrons, and greenhouses. To our knowledge, there has, to date, been no direct comparison of heat tolerance among these cultivars, presumably due to the technical limitations of each method, availability of germplasm, and expected growth response differences of *indica* and *japonica* types under specific screening conditions. Additionally, the optimal conditions for assaying heat-induced spikelet sterility have not been established.

It is generally known that panicles are the rice plant’s heat-sensitive organ [20]. When evaluating cultivars in phytotron assays, one concern was that cultivar-to-cultivar variations of the panicle position potentially exist, which often causes variations in the distances between lamps and panicles. To reproducibly test heat-induced spikelet sterility, we have developed a new custom-built assay system that can be performed in two environmentally controlled growth chambers. In this system, the panicle top position in the artificial paddies that simulate the environmental conditions of the rice paddy (referred to as ‘an artificial rice paddy’) have been made to be adjustable, so that the position of all cultivars/lines can be uniform. We report here the robustness and reproducibility of the assay system by testing both high temperature-induced spikelet sterility and panicle temperature among the reported heat-tolerant rice cultivars.

## Methods

### Plant materials and growth conditions

Rice seeds from nine cultivars (*Oryza sativa* L., see Table 1 for details) were sterilized, incubated in water at 15 °C for 7 days, seeded at 32 °C for 2 days, and grown in a phytotron (Growth Chamber TGE-3CS; Tsubuku Co., Ltd., and AirPEX Engineering Co., Ltd., Kurume, Japan) under metal halide lamps (M150FCLSP2-W/BUD, Iwasaki Electric Co., Ltd., Tokyo, Japan) with an intensity of 850 µmol photons m^−2^ s^−1^ PAR at the canopy position. They were cultivated in a cycle of day/night air temperatures of 26 °C (13 h, 5:50–18:50)/22 °C (11 h, 18:50–5:50) at 60% relative humidity (RH). The air temperature and relative humidity were controlled by using the sensors that placed at the panicle height of rice plant in the phytotron. For the light conditions, half of the lamps were turned off for 1 h at the beginning and end of 13 h of daytime. The water temperature in the artificial rice paddy (see below) was 24.5 ± 1.2 °C (mean ± SD, n = 144), ranging from 22.6 to 26.0 °C. Each plant was grown in a plastic pot (7 cm diameter, 30 cm in height) filled with 1120 ml of rice nursery culture soil containing 0.20 g of nitrogen and 0.12 g each of phosphate and potassium, and each plant was restricted to the main culm by the removal of the tillers. The resulting plants grew under the above conditions until the heading day, which is the day on which a panicle first appears on a rice plant.

**Table 1:** Rice cultivars and genotypes used in this study

Cultivar	Genotype
Akitakomachi (AKT)	*japonica*
Hitomebore (HTM)	*japonica*
Koshihikari (KSH)	*japonica*
Nipponbare (NPB)	*japonica*
Hinohikari (HNH)	*japonica*
Nikomaru (NKM)	*japonica*
IR24	*indica*
IR36	*indica*
N22^a^	*aus*

^a^The seeds of N22 used JP.No.13107 from NARO Genebank

### Heat stress assay system in a phytotron

**Fig. 1:**
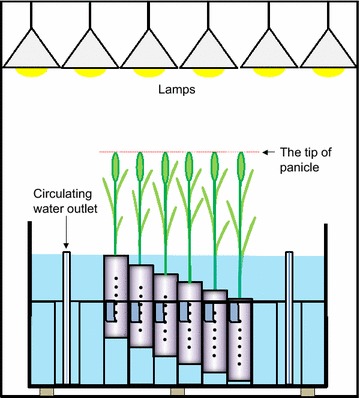
Schematic diagram of the heat tolerant assay system with an artificial rice paddy in the growth chambers. The phytotrons had 24 metal halide lamps in total, and the artificial rice paddy can fit 216 height-adjustable potted plants (diameter 7 cm × height 30 cm), so that the distance between lamps and the tip of panicle could be uniform for all cultivars (see the *dashed line in red* and “Methods”). In the system, water evacuated from outlet (not shown) circulated through the filtration equipment to a water tank placed outdoors using a pump, and was resupplied to the paddy from an outlet after water temperature was controlled

We developed a phytotron assay system for spikelet sterility tolerance (Fig. 1). The phytotron has 24 metal halide lamps built-in and includes an artificial rice paddy (1.33 m × 1.33 m × depth 0.62 m) filled with water (0.51 ton). The water in the artificial rice paddy was circulated through an external flush tank, and returned to the flush tank after filtration with a pump. The artificial rice paddy is capable of containing a maximum of 216 pots with height adjustment to a maximum of 15 cm. To allow for height adjustment, a pair of six holes were punched in 3 cm steps at each of the two sides of each pot (Fig. 1).

### High temperature treatment

On heading day, the potted plants were transferred to another phytotron, and exposed to cycles of 30–36 °C (13 h light)/26–30 °C (11 h dark) at 60% RH for 3 days. The height of the panicle tip was maintained at 70 cm by adjusting the pot from the water surface of the artificial rice paddy each day. For N22, which had a relatively large panicle height, the pot was set to the lowest position, and in case the height became >70 cm the culm was gently leaned over the adjacent pot space to ensure the same panicle tip height (70 cm above the water surface of the artificial rice paddy). The treated plants were then transferred to the previous phytotron and exposed to 26 °C (13 h light)/22 °C (11 h dark) at 60% RH until harvest.

### Measurement of panicle temperature

According to Maruyama et al. [19], a data logger system (CR23X; Campbell Scientific, Inc., USA) was used to measure the spikelet temperature (referred to as ‘panicle temperature’) for one day after inserting a fine wire copper-constantan thermocouple into a spikelet between glumes on the second day of heat treatment. The spikelet was measured at approximately one third of the distance from the tip of the panicle.

### Assay of spikelet fertility

After harvest, the panicles were dried at 30 °C for 3 days, separated into three sections (upper, middle, and under sections) in the primary rachis branches, and the number of mature seeds and empty caryopses of panicles of the middle section were counted.

### Data analysis

Data were subjected to analysis of variance (ANOVA) using JMP software (version 12.1.0, SAS Institute Inc., Cary, NC). Analysis of the spikelet fertility and panicle temperature data was conducted using a Tukey’s Honestly Significant Difference (HSD) test with JMP software.

## Results

### Design of a heat stress assay system in a phytotron

**Fig. 2:**
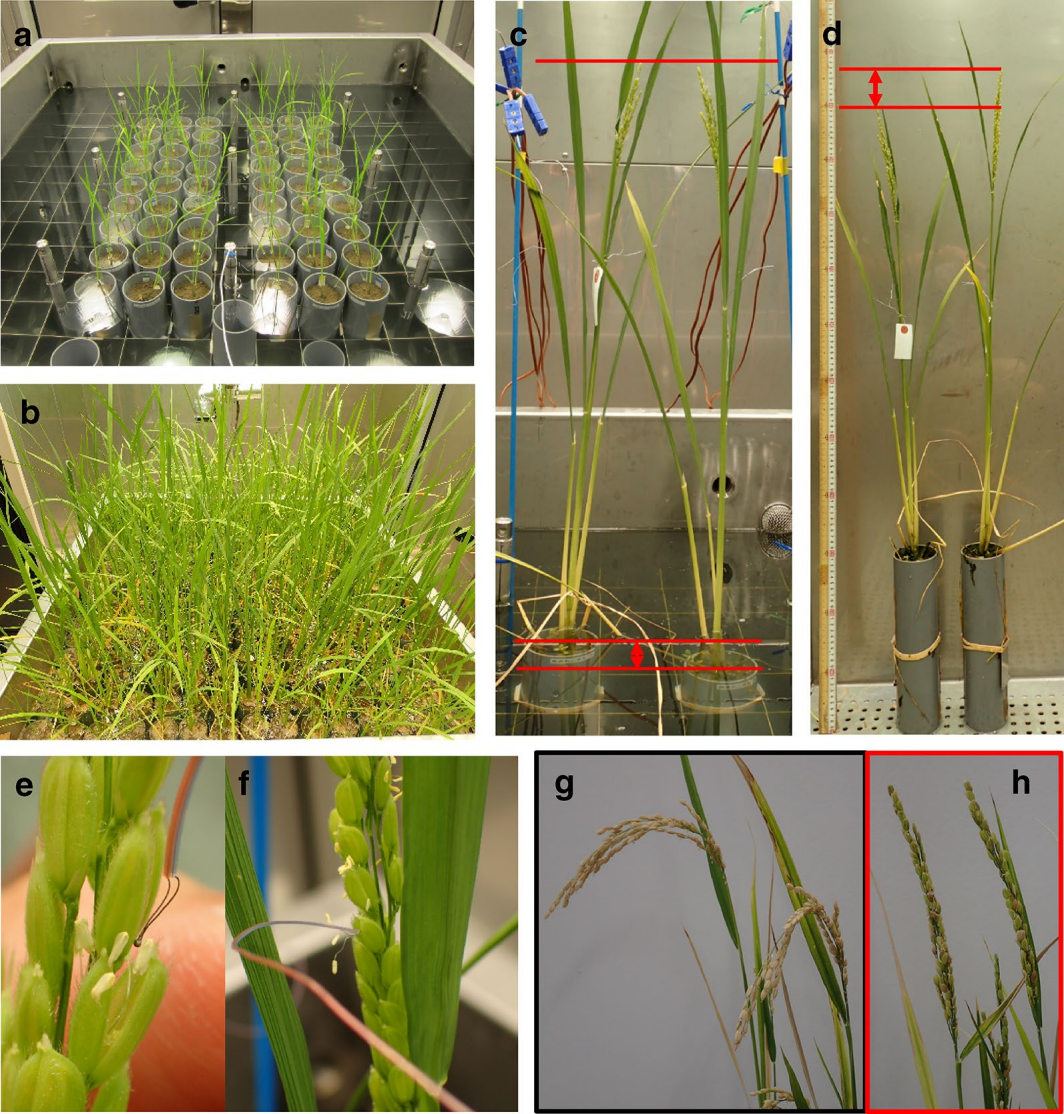
The flow of the assay system of high temperature-induced spikelet sterility. **a** Rice seedlings two weeks after sowing. One seedling was transplanted to a particular pot and grown at 26 °C (13 h light)/22 °C (11 h dark). **b** Rice plants one month after transplantation. **c** Rice plants under heat treatment. The panicle height of rice was fixed 70 cm from the water surface of the artificial rice paddy on heading day. **d** Rice plants after heat treatment. **e** Insertion of a thermocouple into flowering cultivars under heat treatment. **f** Completion of attachment of a thermocouple with a closed flower. **g** Panicles at 40 days after heading day under normal conditions. **h** Panicles at 40 days after high temperature treatment

We provided a rice cultivar, Nipponbare (NPB) as a medium heat tolerance cultivar to reevaluate its rice fertility using the assay system and determine the optimal conditions for detecting the varietal differences reported with various heat tolerant cultivars. Two sets of phytotrons were used; one was used to grow rice to heading at 26 °C (13 h light)/22 °C (11 h dark), and used for grain filling after heat treatment, and the other was used for heat treatment under conditions of 35 °C (13 h light)/29 °C (11 h dark) for 3 days. The rice seedlings were grown for two weeks after germination, and then transferred to individual pots (Fig. 2a). Tillers were removed every 7 days until heading (Fig. 2b). On the day of heading, plants were transferred to another phytotron, and the height of the pots was adjusted each day over 3 days so that the panicle tip was set to 70 cm from the water surface on the artificial rice paddy during the duration of heat treatment (Fig. 2c). Among the plants shown in the photograph, the difference in height of the panicles after treatment was approximately 7 cm (Fig. 2d). Furthermore, during flowering at the 2nd day of heat treatment, a thermocouple was inserted into the floret at approximately one third of the distance from the panicle tip (Fig. 2e). The time course of panicle temperature under the 35 °C/29 °C treatment and water temperature were measured at 10-min intervals. Although the observed panicle temperature was initially unstable after thermocouple insertion, it was stably measured from the next day and was slightly higher than the air temperature during daytime (Additional file 1: Fig. S1). Based on this result, panicle temperature measurement in the floret was possible after the flower closed (Fig. 2f). In the 35 °C/29 °C treatment, the water temperature in the artificial rice paddy was 29.3 ± 1.8 °C (mean ± SD, n = 144), ranging from 26.3 to 32.0 °C (see Additional file 1: Fig. S1). After the heat treatment, the plants were returned to the previous phytotron [26 °C (13 h light)/22 °C (11 h dark)], and ripened for approximately 40 days (Fig. 2h). Additionally, plants grown without high temperature processing were used as the control (Fig. 2g). Under these conditions, the control plants had normal spikelet fertility, in contrast to the high temperature-treated plants which exhibited relatively large spikelet sterility with a high reproducibility.

### Determination of optimized conditions for identifying varietal differences

**Fig. 3:**
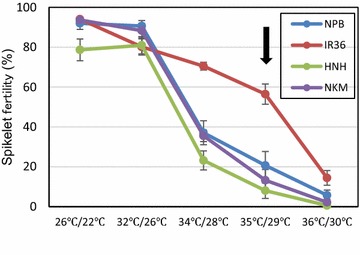
Evaluation of temperature-response causing high temperature-induced spikelet sterility. The chart indicates spikelet fertility for the four rice cultivars [IR36, tolerant; Ninohikari (HNH) and Nikomaru (NKM), sensitive; Nipponbare (NPB), medium tolerance] under each treatment. The *arrowhead* indicates the optimal conditions to assay heat-induced spikelet sterility in this system. The data are the mean ± SE (n = 3–10)

The 3-day high temperature treatment with a series of day/night temperature cycles with four cultivars comprising IR36 (heat-tolerant), NKM and HNH (heat-sensitive), and NPB (medium tolerance) showed a clear varietal difference among cultivars at >34 °C/28 °C. Under the 32 °C/26 °C conditions, there was no varietal difference in high temperature-induced spikelet sterility (Fig. 3). In contrast with the 32 °C/26 °C cycle, the 34 °C/28 °C cycle induced a reduction in spikelet fertility, and cultivar differences observed in the 35 °C/29 °C cycle showed the largest variations in all temperature conditions tested (Fig. 3). However, florets treated at 36 °C/30 °C showed the smallest spikelet fertility with almost no varietal differences. Based on these data, it was suggested that the optimal conditions to assay heat-induced spikelet sterility using this system was 35 °C/29 °C for 3 days.

### Varietal difference of nine heat tolerance cultivars

**Fig. 4:**
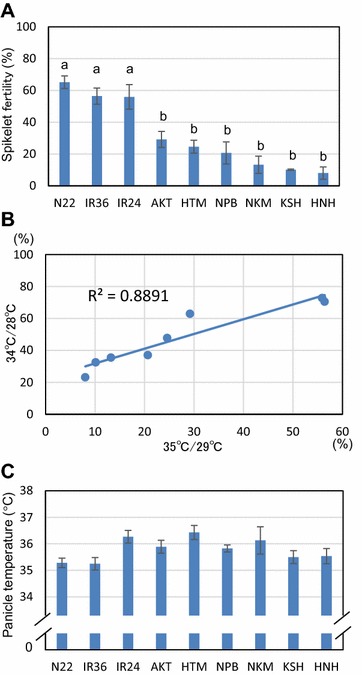
Varietal differences for high temperature-induced spikelet sterility in the phytotron assay system. **A** Spikelet fertility of nine rice cultivars under 35 °C/29 °C for 3 days. **B** Relationship between spikelet fertility at 35 °C/29 °C and at 34 °C/28 °C of eight cultivars except for N22. **C** Panicle temperature of nine rice cultivars under 35 °C/29 °C. Panicle temperature indicated was the average of 10:00–16:00 of stable range. The data are the mean ± SE (n = 3–5). The *same letters* on the chart are not significantly different at the 5% level by the Tukey–Kramer test

We used nine cultivars of rice (*Oryza sativa* L.): three tolerant cultivars, N22, IR24, and IR36; and six popular Japanese cultivars with high palatability, AKT, HTM, KSH, NPB, HNH, and NKM (see Table 1). Under the 35 °C/29 °C treatment, the spikelet fertility of N22 was 65.1% (maximum); that of IR36 was 56.4%, and the least fertile was HNH at 8.0% (Fig. 4A). In the *japonica* rice cultivars, AKT had maximal fertility with 29.2%. In the spikelet fertility rates in eight cultivars except for N22, there was a positive correlation (R^2^ = 0.889) among the two different heat treatment cycles at 35 °C/29 °C and 34 °C/28 °C (Fig. 4B). Moreover, the daytime average panicle temperatures during the 35 °C/29 °C treatment in IR36 and HTM were 35.2 °C and 36.4 °C, respectively (Fig. 4C). There was no significant difference among the nine cultivars for panicle temperature, and there was no positive correlation between heat tolerance and panicle temperature (not shown).

## Discussion

In this study, we have developed a reliable screening system performable in a phytotron for high temperature-induced spikelet sterility in rice (Fig. 1). In this system, providing only the main culm and fixing the distance from the lamp to the panicle allowed us to rule out the potential artifact of cultivar-to-cultivar variations in lamp-to-panicle distances. Consequently, it has been demonstrated that our system was capable for identifying two distinct tolerant groups even among nine tolerant cultivars previously reported.

### The robustness of the developed high temperature-induced spikelet sterility assay system

Consistent with Maruyama et al. [19], we also observed similar varietal differences obtained in the TGC that are required to generate the temperature-versus-fertility curve in each cultivar. Hence, it was concluded that our system is capable of simply but reproducibly evaluating varietal differences of high temperature-induced spikelet sterility without generating any curve in each cultivar. Unlike leaves, the panicle is likely to be a heat-sensitive organ [20], and thus the distance from lamp to panicle should be a crucial factor influencing spikelet fertility. Therefore, it was essential to exclude the potential artifact by keeping the distance between lamps and panicle tips uniform when evaluating potential varietal differences under the artificial light in a phytotron. In the preliminary experiment, ten plants per 1/5000 Wagner pot were transplanted and grown “without removing tillers”, which provided considerably various panicle heights even for the same cultivar in a pot. When the same eight cultivars were examined by fixing the distance between the lamp and the panicle (Additional file 2: Fig. S2A), the evaluation failed. The trend obtained in the preliminary evaluation differed from Maruyama et al. [19] and the results of this work in which AKT, the high tolerance cultivar, had the lowest spikelet fertility, whereas conversely the sensitive cultivar NKM had comparatively high tolerance (Additional file 2: Fig. S2B). This contradiction could be explained by the variation in panicle heights in each pot. Additionally, the treatment of an individual plant at heading was unlikely to be uniform, presumably because of the differences in heading dates of each panicle in one pot, together with the difference in panicle heights among 10 individuals. These issues could be solved by using the newly designed pots with a height adjustment function (see Fig. 1) in the artificial rice paddy even with another potential influence of panicle excursion rate (typically, between 2 and 5 cm/day, see [21]) (Figs. 1, 2). It is true that the optimization of the adjustable height system may be another issue to address prior to the experiment. Although the proposed height adjustment in each cultivar was successfully conducted for three days during the examination and did not require any further adjustment, except for N22 (see “Methods”), it may be necessary to extend the size of height-adjustment, depending on the cultivars.

Maruyama et al. [19] reported eight varietal differences using a temperature gradient chamber (TGC). Their system measured spikelet sterility under various temperature conditions that included a temperature gradient in the chamber, resulting in the detection of varietal differences to heat exposure with accuracy. However, there was a technical limitation for the number of cultivars to be tested, and under natural light, the assay could be influenced by fluctuating environmental conditions (e.g., shade). Additionally, this method requires more time and effort in checking panicle temperature, in addition to the difficulty of temperature management throughout a whole year. In contrast, our phytotron system, presented here, requires less effort and enables a reliable evaluation of heat tolerance among a greater number of candidate cultivars under fixed temperature conditions throughout a year.

### Comparison with tolerance evaluation of previous reports

We revisited the question of whether the results obtained in the indirect TGC method hold true. Tolerance of heat-induced spikelet sterility in eight cultivars including 6 *japonica* and 2 *indica* cultivars used in Maruyama et al. [19] was determined by the temperature (*T*_75_) at which spikelet fertility becomes 75% of that of the control plants. They reported tolerance to spikelet sterility under high temperature was in the order of highest to lowest of; IR36, IR24, AKT, KSH, HTM, NPB, HNH, and NKM. In our study, following the 35 °C/29 °C treatment the tolerance to spikelet sterility was in the order of highest to lowest of; IR36, IR24, AKT, HTM, NPB, NKM, KSH, and HNH, and indicated similar varietal differences to those seen previously, except for the tolerance of KSH (Fig. 4A). They were shown to correlate (R = 0.77, p < 0.05). Several investigators showed that an *aus* cultivar, N22, had very high heat-induced spikelet sterility tolerance [15, 17, 18]. Other tolerant cultivars reported include NPB, AKT [9, 13], and KSH [11]. We evaluated all of these tolerant cultivars at once under high temperature conditions using the current system. Our results showed that the tolerance of N22 was prone to be higher than IR36, and the other cultivars showed similar varietal differences among the eight cultivars used by Maruyama et al. [19] (Fig. 4A), suggesting that this spikelet sterility assay system had high reliability and could be used to identify varietal differences even among cultivars (Figs. 3, 4) in the phytotron as an alternative method of TGC. Further evaluations using other candidate cultivars with high heat tolerance will be of importance in relation to breeding.

Furthermore, it was necessary to identify cultivars with heat tolerance greater than N22 for breeding purposes. The data suggested that the 3-day 35 °C/29 °C treatment allowed us to detect varietal differences even when the tolerance level indicated a numerical value above the 65.1% spikelet fertility of highly tolerant N22 cultivar (Fig. 3). Selecting highly heat tolerant cultivars from our genetic resources is currently under investigation.

### Panicle temperature displays varietal differences

Panicle temperature based on *T*_75_ showed the certain varietal differences from +0.1 °C to −2.5 °C treatment temperatures [19]. In our phytotron assay system, the position of each potted plant was randomized in the artificial rice paddy to minimize possible variations in the set air temperature. The panicle temperature observed in the 35 °C/29 °C treatment showed that the recorded panicle temperatures of all cultivars were slightly higher than the air temperature (Fig. 4C). This difference might be explained by the absorption of the infrared rays from the metal halide lamps in panicles, as Yoshimoto et al. [22] discussed. Time course data during the period from insertion to removal of the sensors indicated relatively stable panicle temperature on the second day even with dynamic changes in water temperature (Additional file 1: Fig. S1), which is consistent with Nishiyama and Satake [20] who reported that the panicle was sensitive to heat. Under the field conditions, certain varietal differences in panicle temperature by evapotranspiration of the community or in panicle height are known to be important characters for high temperature-induced spikelet sterility tolerance [23]. Therefore, one would expect that the 1.2 °C difference might be due to the varietal differences of panicle temperatures; however, it should not be ignored that there was essentially no statistical difference in panicle temperature among cultivars, but there was a clear varietal difference for spikelet fertility, as shown in Fig. 4. Moreover, we did not observe any clear correlation between spikelet sterility tolerance and panicle temperature in this study. This has implied another possibility that high-temperature induced spikelet sterility may be superimposed by several factors including panicle temperature. Our data also suggest that the newly developed system is capable of isolating the influence of panicle temperature. Such a reproducible tolerance assay that could similarly eliminate potential artifacts will be useful in future studies. This may directly lead to the discovery of metabolites or genes that participate in the cause organization of spikelet sterility like the anther or pollen.

## Conclusion

The spikelet sterility caused by high temperatures is expected to become a serious problem in rice globally, including Japan [4]. Thus, it is desirable to breed rice varieties that combine high temperature tolerance with high palatability. We developed a new assay system for high temperature-induced spikelet sterility performable in phytotrons by producing uniform panicle heights in all tested cultivars under heat conditions. The data clearly showed that the system is capable of assaying spikelet sterility in an artificial rice paddy with a high reproducibility throughout a year. Therefore, the use of the system (see Table 2 for details) would allow for a stable and highly accurate evaluation in candidate cultivars/lines. In the future, research into high temperature-induced spikelet sterility will accelerate the following research into heat tolerance in popular cultivars and selection of the most heat tolerant cultivars. Additionally, this system could be used as an analytical tool for the analysis of genetic factors such as transcriptome and metabolome analyses. Finally, it is important to keep in mind that the varietal differences in the tolerance of high temperature-induced spikelet sterility will need to be crosschecked under field conditions, in addition to this phytotron assay. Development of such a system may also provide useful information for screening cultivars/lines in most annual crops including rice to accelerate genetic research.

**Table 2:** A list of the main components of the heat tolerance assay system

Item	Manufacturer/supplier	Type/part no.
Growth chamber	Tsubuku Co., Ltd., Kurume, Japan	TGE-3CS
Artificial rice paddy	Tsubuku Co., Ltd., Kurume, Japan	Width 1.33 m × depth 1.33 m × height 0.62 m, water capacity 510 L
Plastic pot	Tsubuku Co., Ltd., Kurume, Japan	Diameter 7 cm, height 30 cm, 6 holes in 3 cm steps at each of the two sides
Metal halide lamp	Iwasaki Electric Co., Ltd., Tokyo, Japan	M150FCLSP2-W/BUD
Refrigerator	Toshiba Carrier Corp., Kawasaki, Japan	TAM131AM-SV
Temperature control tank	Tsubuku Co., Ltd., Kurume, Japan	Width 0.7 m × depth 0.7 m × height 1.52 m, water capacity 100 L
Circulating pump	Sanso Electric Co., Ltd., Himeji, Japan	PH2-2/2AS6.4

## Additional files

**Additional file 1: Fig. S1.** Time course of changes in set air temperature (*T*_*a*_: solid line), panicle temperatures 1 (*T*_*P*1_: closed circle) and 2 (*T*_*P*2_: opened circle), and water temperature (*T*_*W*_: closed triangle) in the artificial paddy field for 2 days recorded after inserting the fine thermocouples in two spikelets. Panicle temperatures were measured once the flowers were closed, as *T*_*P*1_ and *T*_*P*2_ started at 1 h after insertion of thermocouples. ‘+TC’ and ‘-TC’ indicate the time at insertion and removal of the sensors, respectively. Black bars indicate night. Note that the temperature of water in the paddy displayed diurnal changes according to the changes in air temperature and half of the lamps were turned off for 1 h at the beginning and end of 13 h of daytime, where *T*_*P*1_ and *T*_*P*2_ started to decline prior to the decline in *T*_*a*_.

**Additional file 2: Fig. S2.** The high temperature-induced spikelet sterility assay system before improvement in this study. A. The system had a fixed distance from lamp to panicle. Ten plants were planted in the same pot. B. Spikelet fertility of eight rice cultivars [19] was examined at 36 °C/30 °C for 3 days. Values are the mean ± SE of 3–5 plants.
