# Supplementary figures and images for: Development of a new heat tolerance assay system for rice spikelet sterility
Source: Plant Methods. 2017 May 10;13:34. doi: 10.1186/s13007-017-0185-3 (PMC5424382; doi:10.1186/s13007-017-0185-3)

## Slide 1
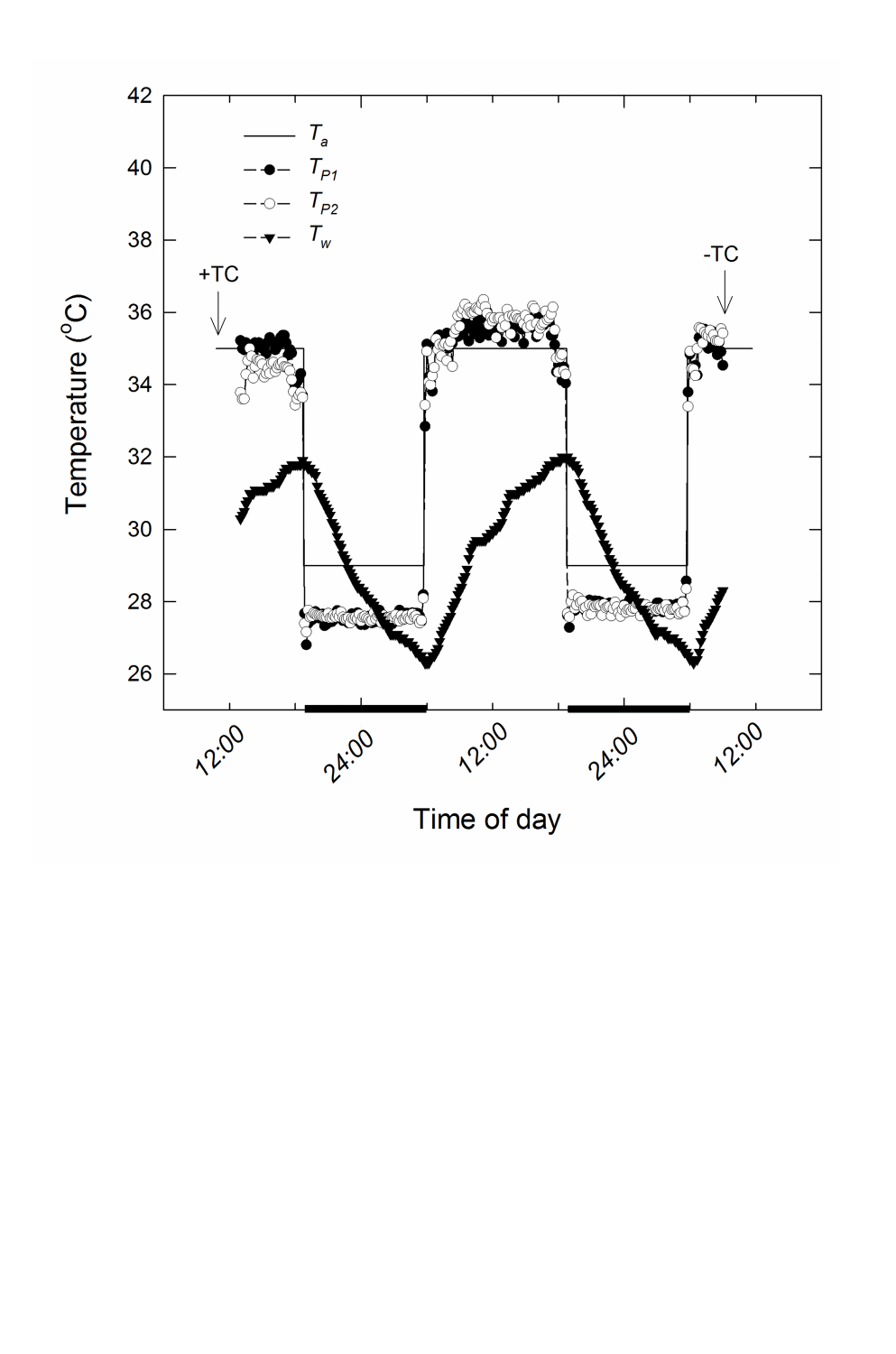

Supplement: Supplementary file 1 — Additional file 1: Fig. S1. Time course of changes in set air temperature (T a: solid line), panicle temperatures 1 (T P1: closed circle) and 2 (T P2: opened circle), and water temperature (T W: closed triangle) in the artificial paddy field for 2 days recorded after inserting the fine thermocouples in two spikelets. Panicle temperatures were measured once the flowers were closed, as T P1 and T P2 started at 1 h after insertion of thermocouples. ‘+TC’ and ‘-TC’ indicate the time at insertion and removal of the sensors, respectively. Black bars indicate night. Note that the temperature of water in the paddy displayed diurnal changes according to the changes in air temperature and half of the lamps were turned off for 1 h at the beginning and end of 13 h of daytime, where T P1 and T P2 started to decline prior to the decline in T a. [file 13007_2017_185_MOESM1_ESM.pptx]

## Slide 1
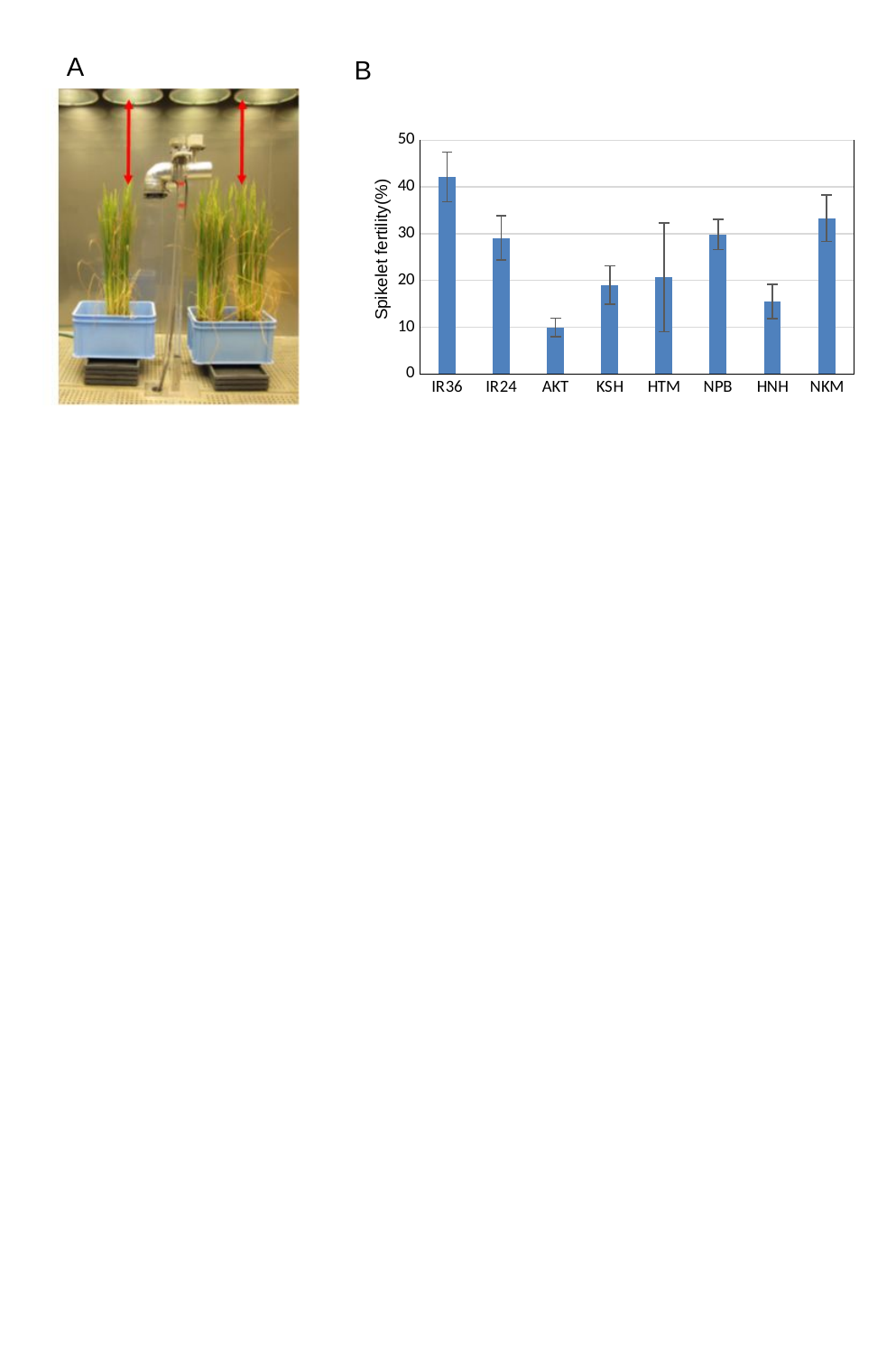

A
B
### Chart
| Category | |
|---|---|
| IR36 | 42.14749543106042 |
| IR24 | 29.124481109731754 |
| AKT | 9.955784038209304 |
| KSH | 19.02617786247732 |
| HTM | 20.650342196071236 |
| NPB | 29.81639202227437 |
| HNH | 15.536757206582488 |
| NKM | 33.35318946663109 |Spikelet fertility(%)

Supplement: Supplementary file 2 — Additional file 2: Fig. S2. The high temperature-induced spikelet sterility assay system before improvement in this study. A. The system had a fixed distance from lamp to panicle. Ten plants were planted in the same pot. B. Spikelet fertility of eight rice cultivars [19] was examined at 36 °C/30 °C for 3 days. Values are the mean ± SE of 3–5 plants. [file 13007_2017_185_MOESM2_ESM.pptx]
